# Supplementary material for: Genome-Wide Differential DNA Methylation and miRNA Expression Profiling Reveals Epigenetic Regulatory Mechanisms Underlying Nitrogen-Limitation-Triggered Adaptation and Use Efficiency Enhancement in Allotetraploid Rapeseed
Source: Int J Mol Sci. 2020 Nov 10;21(22):8453. doi: 10.3390/ijms21228453 (PMC7697602; doi:10.3390/ijms21228453)
Supplement: Supplementary file 1 [file ijms-21-08453-s001.pdf]

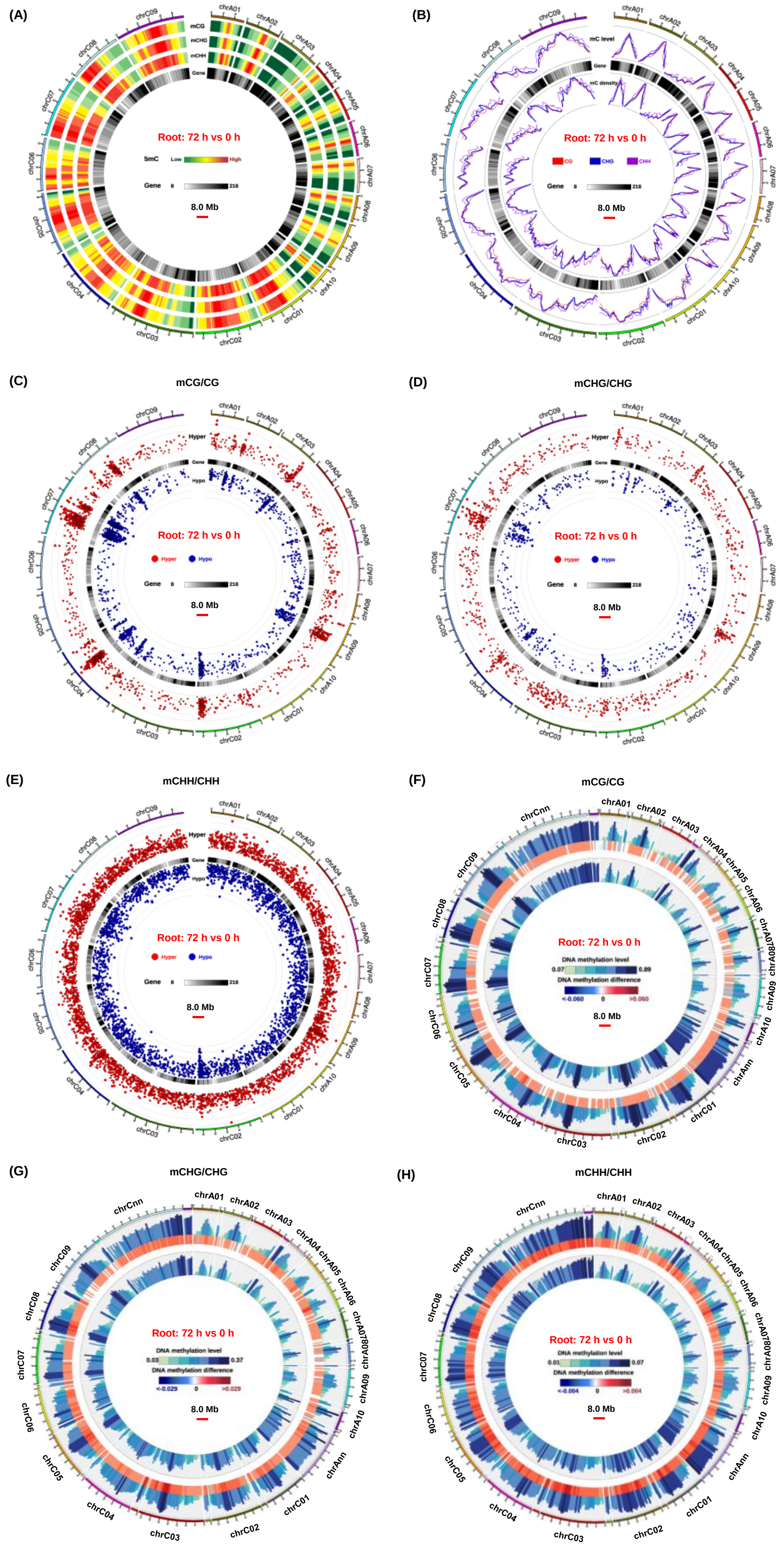
(A)
Root: 72 h vs 0 h
5mC
Low
High
Gene
8.0 Mb
(B)
mC level
mC density
Root: 72 h vs 0 h
CG
CHG
CHH
Gene
8.0 Mb
(C)
mCG/CG
Hyper
Hypo
Root: 72 h vs 0 h
Gene
8.0 Mb
(D)
mCHG/CHG
Root: 72 h vs 0 h
Gene
8.0 Mb
(E)
mCHH/CHH
Root: 72 h vs 0 h
Gene
8.0 Mb
(F)
mCG/CG
Root: 72 h vs 0 h
DNA methylation level
DNA methylation difference
8.0 Mb
(G)
mCHG/CHG
Root: 72 h vs 0 h
DNA methylation level
DNA methylation difference
8.0 Mb
(H)
mCHH/CHH
Root: 72 h vs 0 h
DNA methylation level
DNA methylation difference
8.0 Mb
chrA01
chrA02
chrA03
chrA04
chrA05
chrA06
chrA07
chrA08
chrA09
chrA10
chrAnn
chrC01
chrC02
chrC03
chrC04
chrC05
chrC06
chrC07
chrC08
chrC09
chrCnn

Supplementary Figure S1 Overview of the genome-wide differential DNA methylation fingerprints in the roots of rapeseed plants between N sufficiency and N limitation. (A) Circos plots showing the density of differentially methylated regions across the rapeseed genome. In the Circos figure, the terms were as follows outside-to-inside: (i) chromosome, 5mC density in the CG (ii), CHG (iii), and CHH (iv) contexts, and gene density (v) of each chromosome in alloteraploid rapeseed. (B) Density and level plot of 5mC in CG, CHG, and CHH contexts in the gene bodies on each chromosome. In the Circos figure, the terms were as follows outside-to-inside: (i) chromosome, (ii) 5mC levels in the CG, CHG, and CHH contexts, (iii) gene density, and (iv) 5mC densities in the CG, CHG, and CHH contexts in the gene bodies. (C-E) Density of hyper-/hypo-methylated regions in the CG (C), CHG (D), and CHH (E) contexts across the rapeseed genome. In the Circos figure, the terms were as follows outside-to-inside: (i) chromosome, (ii) densities of hyper-methylated regions in the CG, CHG, and CHH contexts, (iii) gene density, and (iv) densities of hypo-methylated regions in the CG, CHG, and CHH contexts. (F-H) Differential methylation levels in the CG (F), CHG (G), and CHH (H) contexts across the rapeseed genome. In the Circos figure, the terms were as follows outside-to-inside: (i) chromosome, (ii) hyper-methylation levels in the CG, CHG, and CHH contexts, (iii) DNA methylation differences, and (iv) hyper-methylation levels in the CG, CHG, and CHH contexts. The time of 0 h and 72 h refers to the time after treatment of N deficiency.

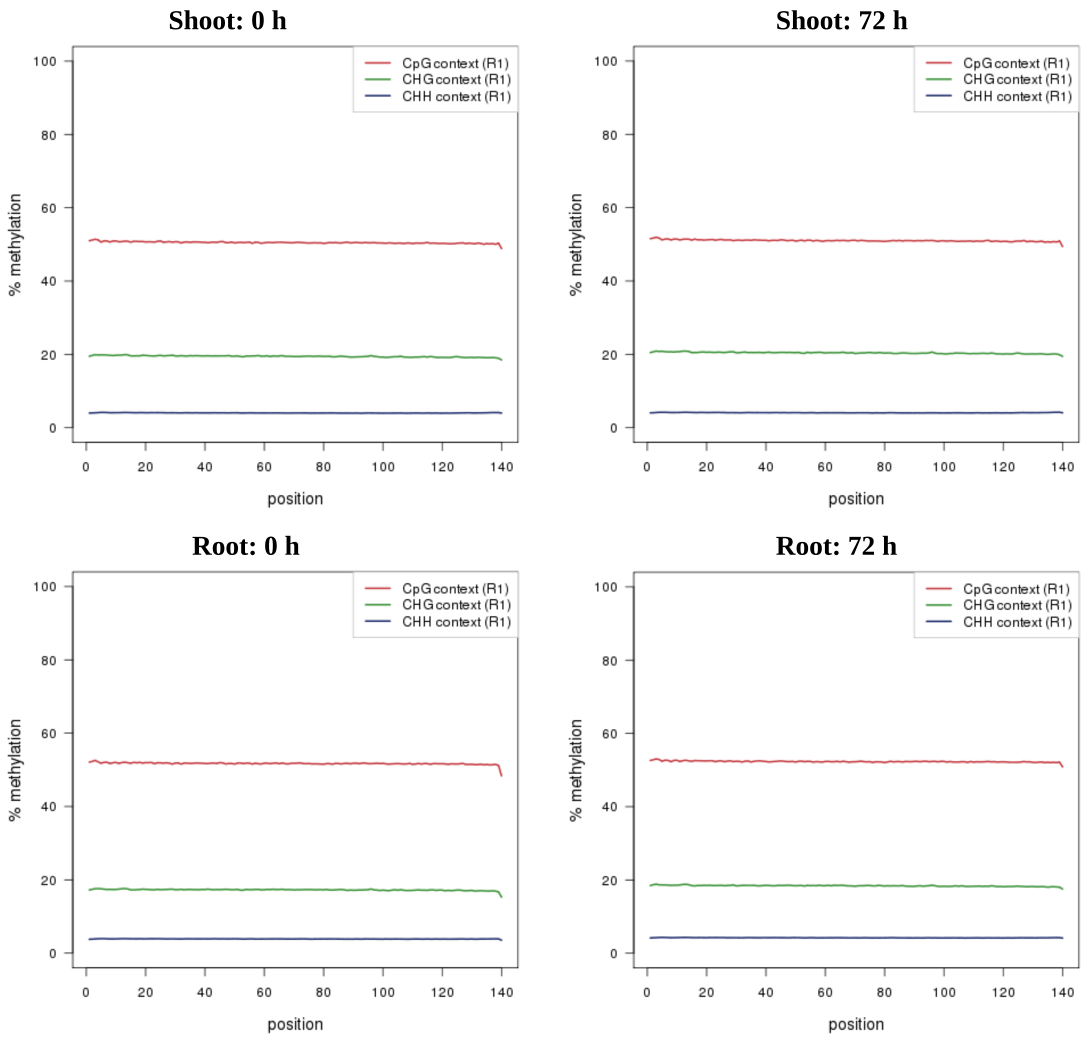

Supplementary Figure S2 Genome-wide DNA methylation (CG, CHG and CHH) levels in the shoots and roots of rapeseed plants under both N sufficiency (0 h) and N limitation (72 h). The time of 0 h and 72 h indicate the time (hour) after N limitation treatment. The X axis indicates the nucletide postion of sequencing reads.

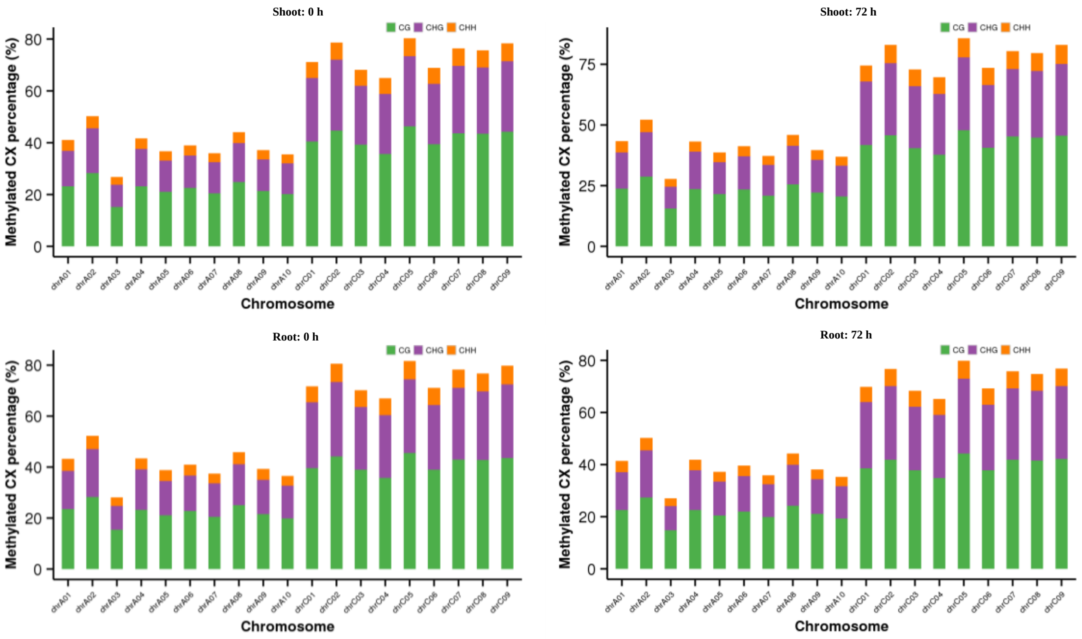

Supplementary Figure S3 Genome-wide DNA methylation (CG, CHG and CHH) levels of each chromosome in the shoots and roots of rapeseed plants under both N sufficiency (0 h) and N limitation (72 h). The time of 0 h and 72 h indicate the time (hour) after N limitation treatment.

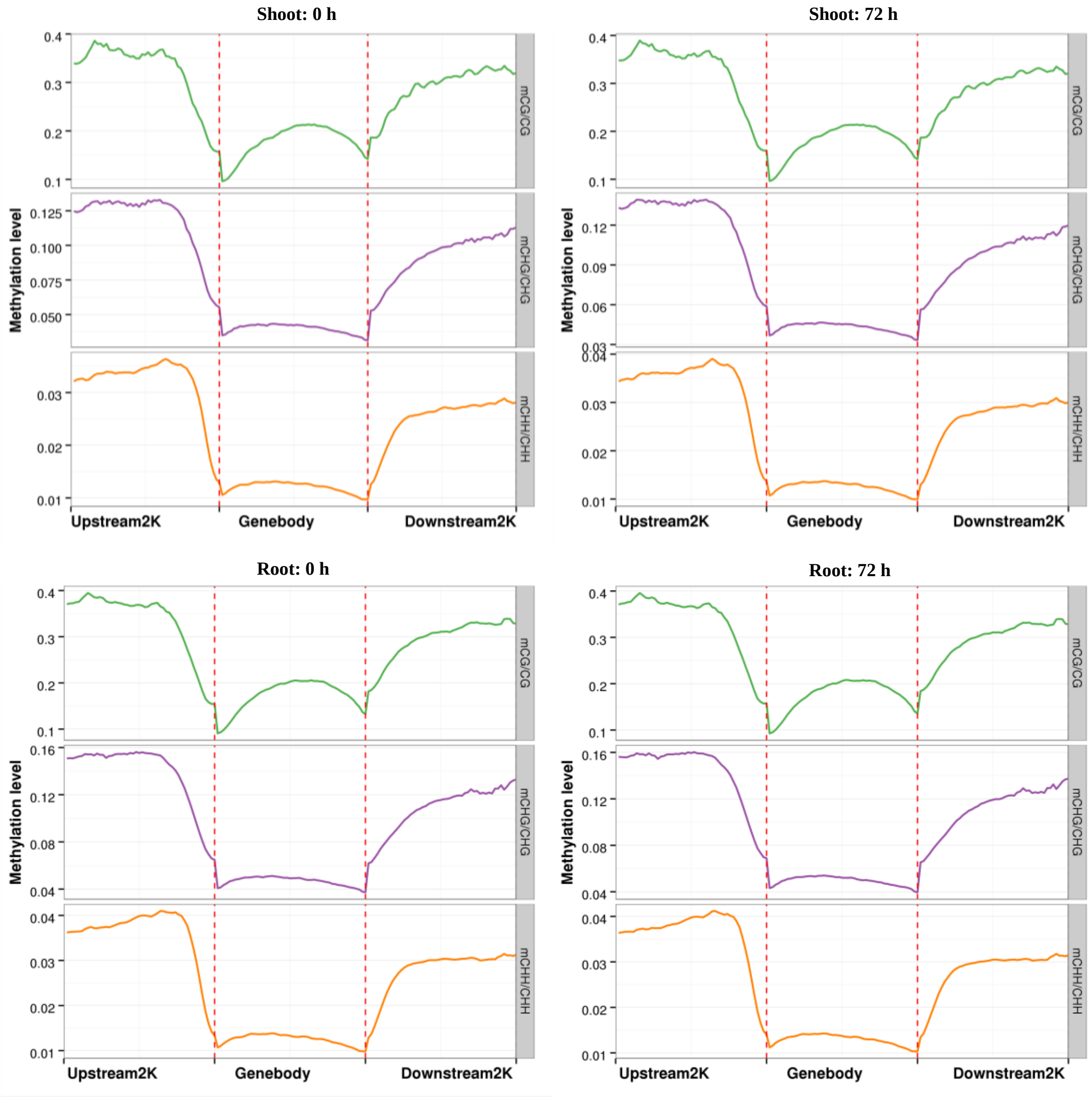

Supplementary Figure S4 DNA methylation levels of gene promoter (2.0 kb region upstream of the transcription starting site), gene body and gene downstream regions in the shoots and roots of rapeseed plants under N sufficiency (0 h) and N limitation (72 h). The time of 0 h and 72 h indicate the time (hour) after N limitation treatment.

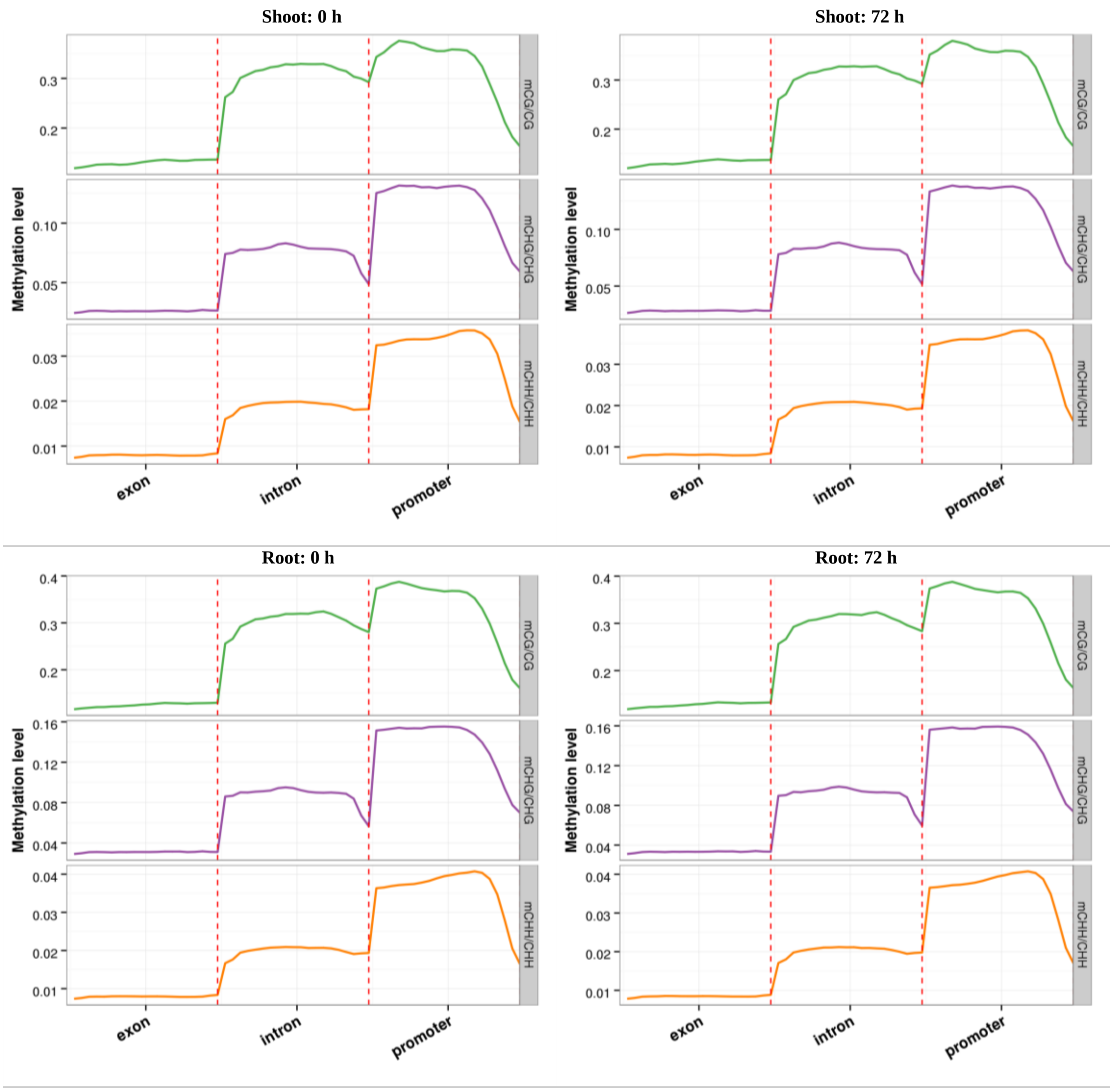

Supplementary Figure S5 DNA methylation levels of gene promoter (2.0 kb region upstream of the transcription starting site) and gene body (mainly exon and intron) in the shoots and roots of rapeseed plants under N sufficiency (0 h) and N limitation (72 h). The time of 0 h and 72 h indicate the time (hour) after N limitation treatment.

(A)

(B)

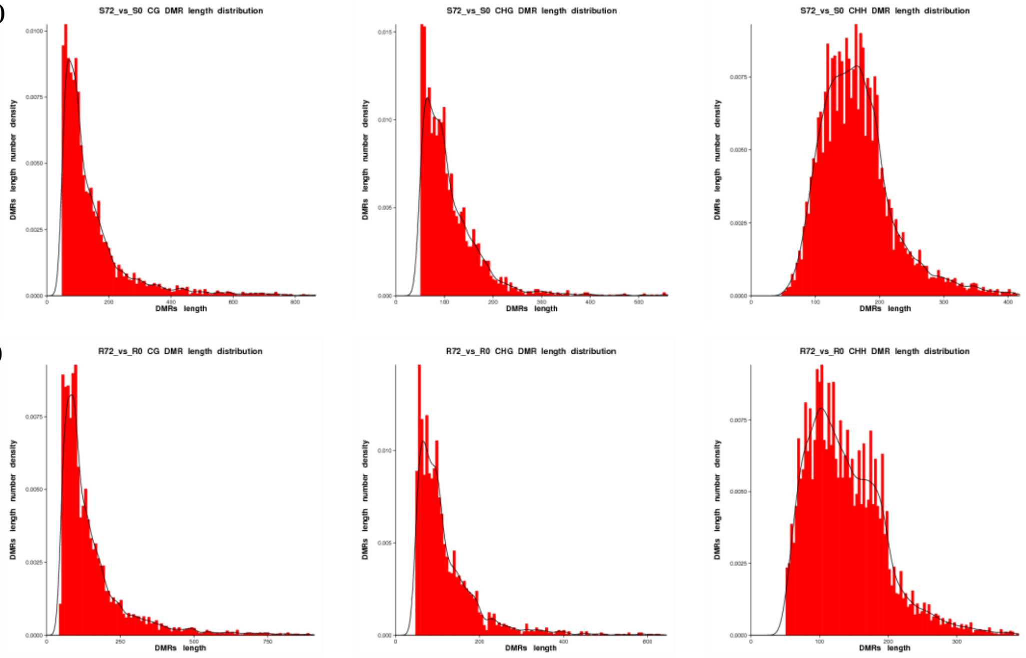

Supplementary Figure S6 Length distribution of differentially methylated regions (DMRs) between N sufficiency and N limitation in the shoots and roots. (A-B) Length distribution of DMRs in CG, CHG and CHH genomic contexts between N sufficiency and N limitation in the shoots (A) and roots (B) of rapeseed plants under N sufficiency (0 h) and N limitation (72 h). S, shoot; R, root. The time of 0 h and 72 h indicate the time (hour) after N limitation treatment.

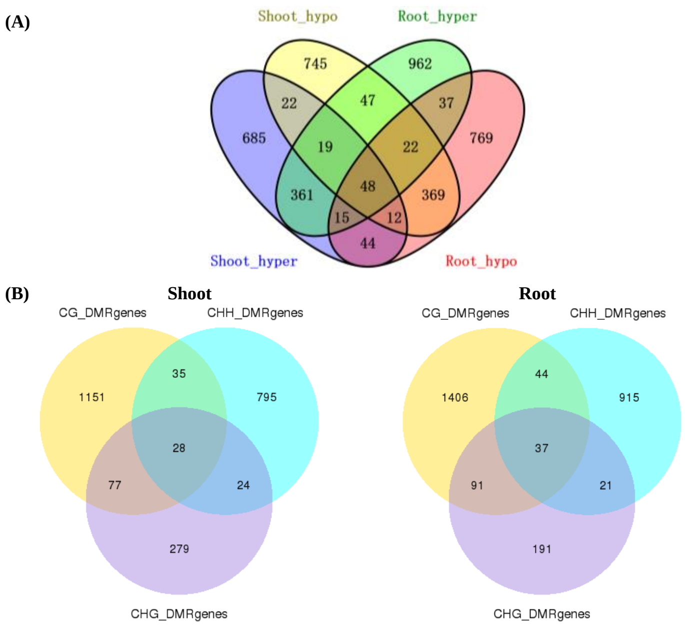

Supplementary Figure S7 Venn diagrams showing hyper-methylation and hypo-methylation of differentially methylated region-associated genes (DMGs) in the shoots and roots of rapeseed plants under N sufficiency and N limitation.

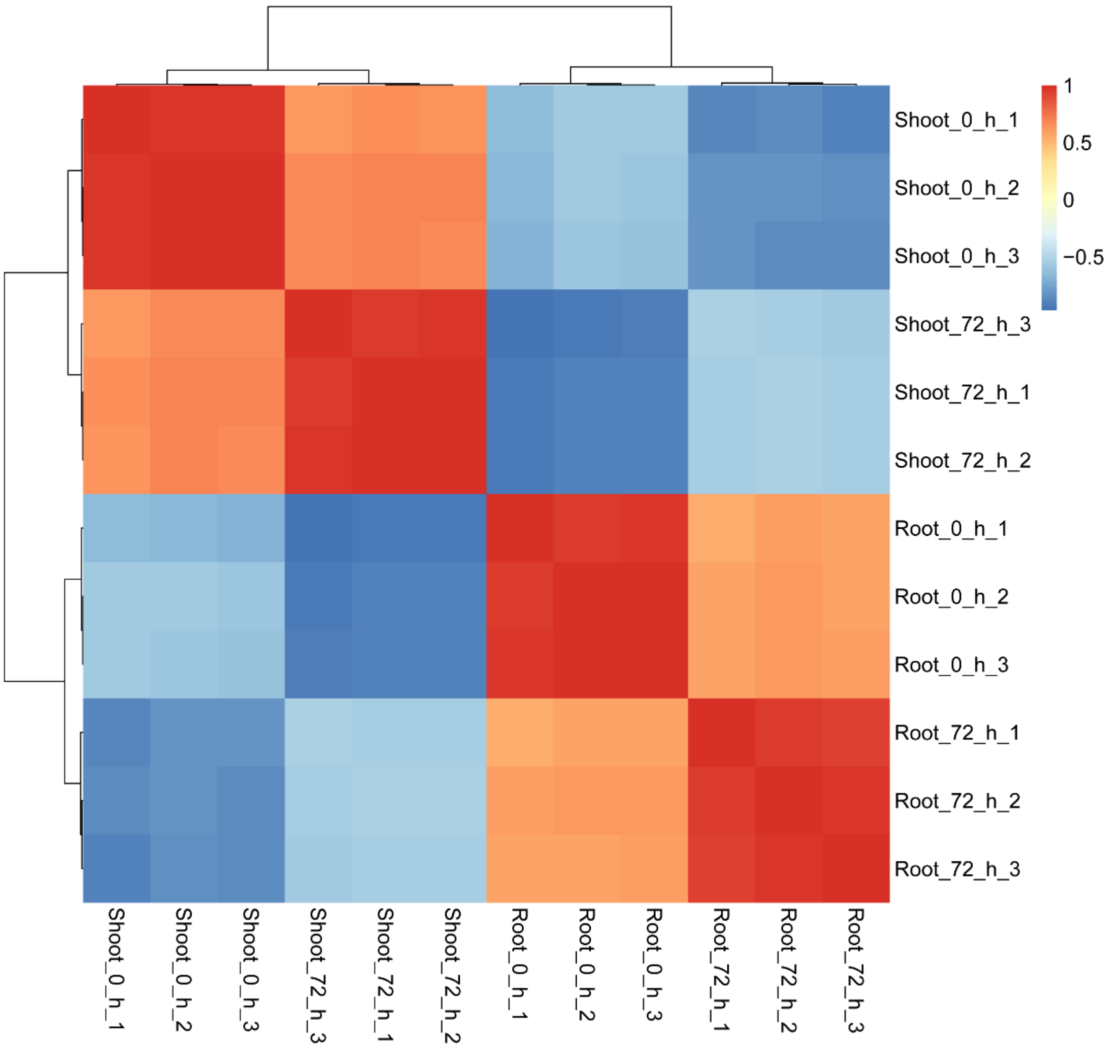

Supplementary Figure S8 *Pearson* coefficients showing the correlation between different samples or biological replicates of each sample in the miRNA sequencing. Each sample of shoots or roots contains three independent biological replicates. The time of 0 h and 72 h indicate the time (hour) after N limitation treatment.

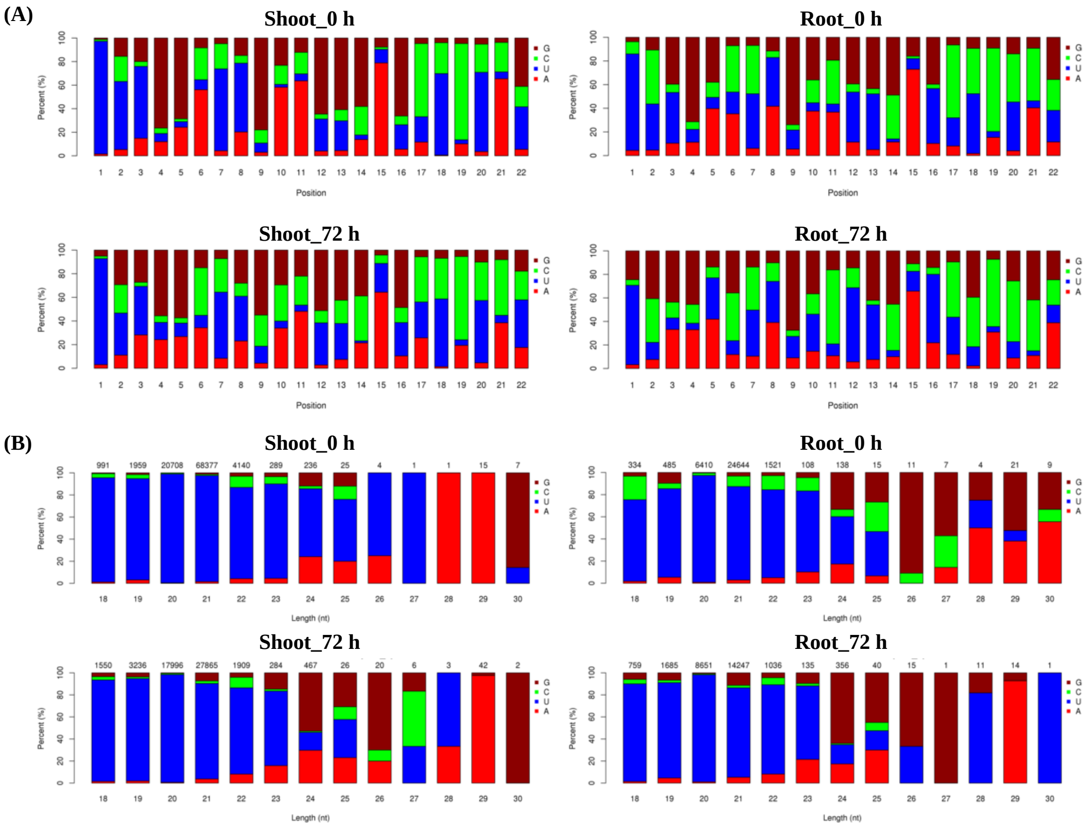

Supplementary Figure S9 Relative nucleotide bias of miRNAs identified in this study. (A) Relative nucleotide bias for each position of miRNAs; (B) relative nucleotide (nt) bias for the 18-30 nt long miRNAs. The time of 0 h and 72 h indicate the time (hour) after N limitation treatment.

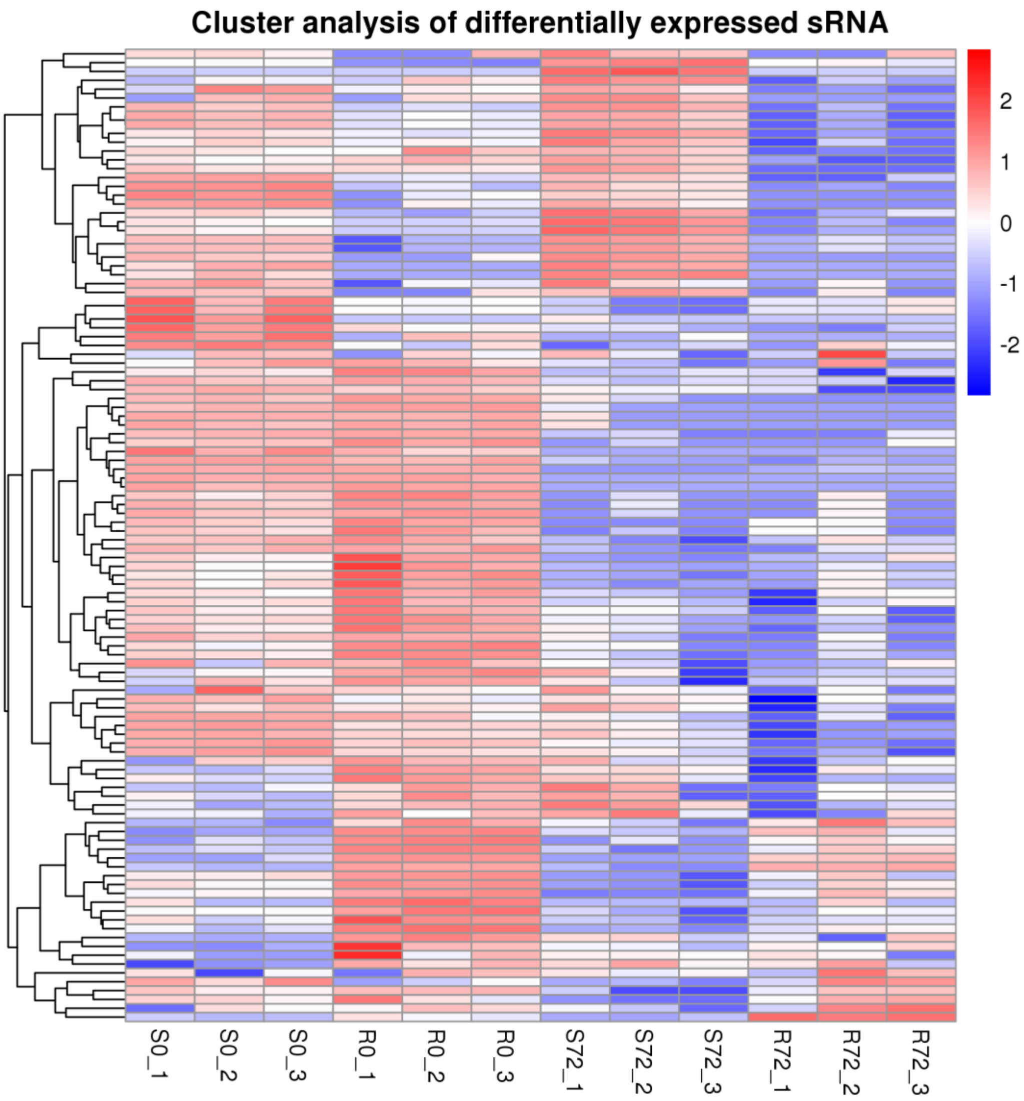

Supplementary Figure S10 Hierachy clustering showing expression profiling of genome-wide miRNAs in the shoots and roots of rapeseed plants under N sufficiency and N limitation. S, shoot; R, root. The time of 0 h and 72 h indicate the time (hour) after N limitation treatment.

Supplementary Tables

Supplementary Table S1 Overview of the whole-genome bisulfite sequencing data

Sample name	Raw reads (Million)	Raw bases (Gb)	Clean reads	Clean bases (Gb)	Clean ratio (%)	Q20 (%)	Q30 (%)	GC content (%)	BS conversion rate (%)
Shoot_0 h	120	36	118625109	32.71	90.87	97.75	92.61	21.39	99.75
Shoot_72 h	120	36	118240208	32.59	90.53	97.63	92.27	21.38	99.78
Root_0 h	120	36	118545047	32.72	90.88	97.77	92.59	21.97	99.75
Root_72 h	120	36	118573825	32.73	90.93	97.80	92.75	22.58	99.74

Supplementary Table S2 Overview of the miRNA sequencing data

Sample	Raw reads	Clean reads	Data size (Gb)	Error rate	Q_{20}	Q_{30}	GC content
Shoot_0 h	17,980,501	17,586,857 (97.81%)	0.90	0.0001	0.98	0.95	49.05
Root_0 h	17,122,378	16,409,800 (95.85%)	0.86	0.0001	0.97	0.95	0.51
Shoot_72 h	16,553,428	16,176,768 (97.73%)	0.83	0.0001	0.98	0.95	0.51
Root_72 h	17,386,670	17,081,876 (98.25%)	0.87	0.0001	0.98	0.96	0.52

Supplementary Table S3 Some identified miRNAs and their mature sequences

miRNA name	Mature sequence (5'-3')	miRNA name	Mature sequence (5'-3')	miRNA name	Mature sequence (5'-3')
Bna-miR1140	ACAGCCUAAACCAAUCGGAGC	Bna-miR172b	GGAAUCUUGAUGAUGCUGCAU	Bna-miR171g	UGAUUGAGCCGCGCCAAUAUCU
Bna-miR156a	UGACAGAAGAGAGUGAGCACA	Bna-miR172d	AGAAUCUUGAUGAUGCUGCAG	Bna-miR172a	AGAAUCUUGAUGAUGCUGCAU
Bna-miR156b	UUGACAGAAGAUAGAGAGCAC	Bna-miR2111a	GUCCUCGGGAUGCGGAUUACC	Bna-miR860	UCAAUACAUUGGACUACAUAU
Bna-miR156d	UGACAGAAGAGAGUGAGCAC	Bna-miR2111b	UAAUCUGCAUCCUGAGGUUUA	Bna-miR169m	UGAGCCAAAGAUGACUUGCCG
Bna-miR159	UUUGGAUUGAAGGGAGCUCUA	Bna-miR2111b	AUCCUCGGGAUACAGAUUACC	Bna-miR169n	CAGCCAAGGAUGACUUGCCGG
Bna-miR160a	UGCCUGGCUCCCUGUAUGCCA	Bna-miR2111c	UAAUCUGCAUCCUGGGGUUUA	Bna-miR6034	UCUGAUGUAUAUAGCUUUGGG
Bna-miR161	UCAAUGCACUGAAAGUGACUA	Bna-miR390a	AAGCUCAGGAGGGAUAGCGCC	Bna-miR6035	UGGAGUAGAAAAUGCAGUCGU
Bna-miR162a	UCGAUAAACCUGUGCAUCCAG	Bna-miR393	UCCAAAGGGAUCGCAUUGAUC	Bna-miR171a	UUGAGCCGUGCCAAUAUCACG
Bna-miR164a	UGGAGAAGCAGGGCACGUGCA	Bna-miR394a	UUGGCAUUCUGUCCACCUCC	Bna-miR171f	UGAUUGAGCCGCGCCAAUAUC
Bna-miR164b	UGGAGAAGCAGGGCACGUGCG	Bna-miR395a	CUGAAGUGUUUGGGGGAACUC	Bna-miR6036	AUAGUACUAGUACUUGCAUGAUCA
Bna-miR166a	UCGGACCAGGCUUCAUUCCCC	Bna-miR395d	CUGAAGUGUUUGGGGGGACUC	Bna-miR824	UAGACCAUUUGUGAGAAGGGA
Bna-miR166f	UCGGACCAGGCUUCAUCCCCC	Bna-miR397a	UCAUUGAGUGCAGCGUUGAUGU	Bna-miR169g	UAGCCAAGGAUGACUUGCCUGC
Bna-miR167a	UGAAGCUGCCAGCAUGAUCUAA	Bna-miR399a	UGCCAAAGGAGAUUUGCCCGG	Bna-miR169c	UAGCCAAGGAUGACUUGCCUA
Bna-miR167c	UGAAGCUGCCAGCAUGAUCUA	Bna-miR399c	UGCCAAAGGAGAUUUGUCCGG	Bna-miR6031	AAGAGGUUCGGAGCGGUUUGAAGC
Bna-miR167d	UGAAGCUGCCAGCAUGAUCU	Bna-miR403	UUAGAUUCACGCACAAACUCG	Bna-miR6032	UGGAGCAUCAACAGAUCUCGG
Bna-miR168a	UCGCUUGGUGCAGGUCGGGAA	Bna-miR6028	UGGAGAGUAAGGACAUUCAGA	Bna-miR6030	UCCACCCAUACCAUACAGACCC
Bna-miR168b	UCGCUUGGUGCAGGUCGAGAA	Bna-miR6029	UGGGGUUGUGAUUUCAGGCUU	Bna-miR169a	CAGCCAAGGAUGACUUGCCGA

Supplementary Table S4 Overview of the genome-wide miRNA expression abundances

Sample	Expression interval*	Proportion (%)
	<15	14.2
Shoot_0 h	15-60	12.88
	>60	72.92
	<15	26.14
Shoot_72 h	15-60	15.34
	>60	58.52
	<15	15.34
Root_0 h	15-60	7.96
	>60	76.70
	<15	33.53
Root_72 h	15-60	14.96
	>60	51.51

*Note: The TPM values of miRNAs that are < 15, 15-60 and > 60 were defined as low, intermediate and high expression intervals.

Supplementary Table S5 Number of transcript reads obtained from the degradome sequencing data

Sample	Root	Shoot
Raw reads	20,228,940	23,667,051
Reads < 15 nt after removing 3' adaptor	97,279	106,709
Clean reads	20,131,661	23,560,342
Transcript mapped reads	14,074,977	18,370,535

Supplementary Table S6 Target gene number of conserved miRNAs by software prediction and degradome sequencing in *Brassica napus*

miRNA_name		Predicted targets transcript	Degradome_Detected	miRNA_name		Predicted targets transcript	Degradome_Detected
miR1140	bna-miR1140	3	1	miR171	bna-miR171g	12	11
miR156	bna-miR156a	62	31	miR172	bna-miR172a	48	23
	bna-miR156b	47	27		bna-miR172b	36	22
	bna-miR156d	85	32		bna-miR172d	61	23
miR159	bna-miR159	30	8	miR2111	bna-miR2111a	2	0
miR160	bna-miR160a	14	14		bna-miR2111b	5	0
miR161	bna-miR161	33	12		bna-miR2111b	11	6
miR162	bna-miR162a	10	2		bna-miR2111c	8	4
miR164	bna-miR164a	27	15	miR390	bna-miR390a	15	4
	bna-miR164b	36	20		bna-miR393	25	17
miR166	bna-miR166a	22	17	miR394	bna-miR394a	24	7
	bna-miR166f	19	17	miR395	bna-miR395a	18	16
miR167	bna-miR167a	8	7		bna-miR395d	15	14
	bna-miR167c	14	9	miR397	bna-miR397a	11	3
	bna-miR167d	34	16	miR399	bna-miR399a	7	2
miR168	bna-miR168a	5	5	miR403	bna-miR403	5	3
	bna-miR168b	7	1	miR6028	bna-miR6028	15	0
miR169	bna-miR169a	13	8	miR6029	bna-miR6029	11	2
	bna-miR169c	16	9	miR6030	bna-miR6030	27	19
	bna-miR169g	17	9	miR6031	bna-miR6031	3	0
	bna-miR169m	11	2	miR6034	bna-miR6034	7	1
	bna-miR169n	21	15	miR6035	bna-miR6035	20	3
miR171	bna-miR171a	11	10	miR824	bna-miR824	13	7
	bna-miR171f	12	11	miR860	bna-miR860	8	1

Supplementary Table S7 Gene-specific primers used for RT-qPCR assays in this study

Gene name	Forward (5′-3′)	Reverse (5′-3′)	Amplification efficiency
NRT1.1	CAAGAAGTTGATTGGTAGCCCG	GTCCTTTATCGCTGCTTTGTCC	99.32%
NRT2.1a	AAAGGTACTGAGGAGCACTATTATGG	GTATTCTGAGGCGGCGTAGC	96.37%
NAR2.1	TCAAGAAGCTCCTTTTCGCG	TAAATTCAGGCTCCTTTGTAGCC	97.64%
NRT1.5	GATGAAGTCACGCCTTGCG	GCAATGTTCGGTTGGTAACCC	97.53%
NRT1.8	GGGTATGGTGGTTATCAGCCC	CGAAAGGAGCGATCCGAGG	96.30%
CLCa	CAGCAACATGCATTAGCAAAGAC	CAAAAGGGATGCTACATGGC	92.9%
NIA1	GTTATTTTTTTCGCTGGAATGAG	AACTTGTGTAAAACGCTATCTTTAACG	97.4%
GLn1;1	CGGGATGACTCCCATGTCG	GATGCTTCTCATGTGTTCAGGG	96.7%
BnaC06g23520D	GCTGGATCATCGGCTTTAGC	GAGCTTCATCTTTCGCTTCTTG	99.7%
BnaEF1α	GCCTGGTATGGTTGTGACCT	GAAGTTAGCAGCACCCTTGG	100.00%
BnaGDI1	GAGTCCCTTGCTCGTTTCC	TGGCAGTCTCTCCCTCAGAT	93.10%
